# Analysis of the Expression of Neurotrophins and Their Receptors in Adult Zebrafish Kidney

**DOI:** 10.3390/vetsci9060296

**Published:** 2022-06-15

**Authors:** Pietro Cacialli, Carla Lucini

**Affiliations:** 1 Department of Pathology and Immunology, School of Medicine, University of Geneva, Rue Michel-Servet 1, 1206 Geneva, Switzerland; 2 Department of Veterinary Medicine, University of Naples Federico II, 80138 Naples, Italy; lucini@unina.it

**Keywords:** zebrafish, kidney, neurotrophin, Trk receptor

## Abstract

Neurotrophins and their receptors are involved in the development and maintenance of neuronal populations. Different reports have shown that all neurotrophin/receptor pathways can also play a role in several non-neuronal tissues in vertebrates, including the kidney. These signaling pathways are involved in different events to ensure the correct functioning of the kidney, such as growth, differentiation, and regulation of renal tubule transport. Previous studies in some fish species have identified the neurotrophins and receptors in the kidney. In this study, for the first time, we compare the expression profiles (mRNA and protein) of all neurotrophin/receptor pathways in the kidney of the adult zebrafish. We quantify the levels of mRNA by using qPCR and identify the expression pattern of each neurotrophin/receptor pathway by in situ hybridization. Next, we detect the proteins using Western blotting and immunohistochemistry. Our results show that among all neurotrophins analyzed, NT-3/TrkC is the most expressed in the glomerule and tubule and in the hematopoietic cells, similar to what has been reported in the mammalian kidney.

## 1. Introduction

Neurotrophins include brain-derived neurotrophic factor (BDNF), nerve growth factor (NGF), NT-3, NT-4/5, and, only in fish, NT 6/7 [1,2,3,4,5,6,7]. The binding to three distinct tyrosine kinase receptors, TrkA, TrkB, and TrkC, mediates their activities concerning neuronal proliferation [8], survival [9,10], differentiation, and plasticity [11] in many neuronal populations of the central and peripheral nervous systems in different vertebrates [12,13,14,15,16]. In mammals, as in fish, besides this essential role, neurotrophins and receptors are also important in non-neuronal cells, as suggested by their detection in different organs [17,18,19,20,21,22,23], including the kidney [24,25,26]. Neurotrophins and receptors are involved in different events to ensure the correct functioning of the kidney, such as growth, differentiation, and regulation of renal tubule transport. Indeed, recent functional in vivo studies, in mice models and in vitro in human patients, have revealed a close association between neurotrophin/receptor pathways and kidney diseases [27,28,29]. However, further studies using emerging animal models [30], such as zebrafish (*Danio rerio*), could be useful to elucidate the specific mechanisms of these pathologies. Zebrafish is a teleost fish of the family of Cyprinidae. This fish presents different characteristics, making it an excellent animal model. The most important advantages are the small size (reduction in maintenance costs and spaces to be used), high fecundity, and rapidity of embryonic development [31]. This fascinating model can be utilized in mutagenesis analyses and drug screening tests [32,33,34]. It has become a popular model organism used to conduct experiments related to different research fields, such as embryology, genetics, cancer, cardiovascular, and organ and tissue regeneration [35,36,37,38]. To study the anatomy, cellular biology, and molecular mechanisms of the vertebrate kidney, the zebrafish is an excellent model [39,40,41,42,43].

The zebrafish kidney is located in the retroperitoneal area. It consists of two different regions, the head and trunk [44,45,46]. Zebrafish and mammalian kidneys have several similarities at the microscopic level; both have nephrons with a glomerulus, proximal tubules, distal tubules, and collecting ducts. The renal interstitium of the zebrafish contains hematopoietic cells [47,48,49,50,51]; thus, it could be considered equivalent to the bone marrow in mammals [52,53,54]. During development, the hematopoietic stem progenitor cells (HSPCs) emerge from the dorsal aorta, colonize the caudal hematopoietic tissue (equivalent to the mammalian fetal liver), and migrate to the kidney marrow [55,56].

To our knowledge, there are no investigations concerning the distribution of mRNA and proteins of neurotrophic factors and their receptors in the adult zebrafish kidney. Therefore, the purpose of the present report is to gain insights into the anatomical localization of neurotrophins and their high-affinity Trk receptors in the kidney of the zebrafish to inspire further experimental investigations in a non-mammal model. This study was carried out using different approaches. We used qPCR and Western blotting, respectively, to quantify the expression of mRNA and proteins in the kidney. Next, we used in situ hybridization and immunohistochemistry, to localize the distribution of all neurotrophins and receptors in the adult zebrafish kidney.

## 2. Materials and Methods

### 2.1. Animals and Kidney Dissection

All procedures were approved by the Institutional Committee of the University of Naples Federico II (CNR, n°2/2020-PR), and according to the Italian Decree 26/2014. Adult females and males (1 year) of *Danio rerio* zebrafish were anesthetized by 0.1% ethyl 3-aminobenzoate and methanesulfonate (Sigma Chemicals Co., St. Louis, MO, USA), and kidneys were excised.

### 2.2. qPCR

From 15 kidneys, the total RNA was extracted using the RNeasy minikit (Qiagen, Frankfurt, Germany) and reverse-transcribed into cDNA using a Superscript kit (Invitrogen, Waltham, MA, USA). Quantitative real-time PCR (qPCR) was performed using the Universal qPCR Kit (KAPA BIOSYSTEMS, Wilmington, DC, USA) and run on a CFX connect real-time system (Bio-Rad, Tokyo, Japan), Fast Sybr Green (Universal Bioline). All data indicate the expression relative to *ef1a*, calculated by [2Ct(*geneX*)-Ct(*ef1a*)]. Each qPCR experiment was performed using biological triplicates. No difference in expression between females and males was found. For experiments using qPCR, each *n* represents the average of biological triplicates from a single experiment. Experiments were repeated at least three times. The primers used have also been described in previous studies [20,57,58,59,60]. Primers used:
*bdnf*F: CGAGGAATAGACAAGCGGCA;R: ATCCGTATAAACCGCCAGCC*ngf*F: GAGAAGACTACAAGCGAAT;R: CGACAACAATAAGGAGGAT*nt3*F: CCCATCAGTGCGCTCATC;R: TCCGAACTGTCCACCATG*nt4*F: GCTCCTCCTAGAACAGAGGG;R: CGTCCTGGATGCATCTTCTCTG*trka*F: GCATTTACAATGGCAGCCAG;R: CTTCTTGAGTGGTCACTGTC*trkb*F: TCACCTATGGCAAGCAACCC;R: CTTTGGGGCAAGTACGAGGT*trkc*F: CGGAAGTGGATTGGACAGTT;R: CATGAAGCCGTTATCGTCC*ef1a*F: TCAAGGACATCCGTCGTGGTA;R: ACAGCAAAGCGACCAAGAGG

### 2.3. Statistical Analysis

Statistical analysis was completed using a one-way ANOVA, adjusted for multiple comparisons; center values denote the mean, and error values denote s.e.m. The software GraphPad Prism (version 7.0a) has been used for all statistical analysis.

### 2.4. Chromogenic and Fluorescence In Situ Hybridization (ISH)

For the preparation of digoxigenin (DIG)-labeled antisense riboprobes, the protocol has also been described in previous studies [13,55]. In detail, *full-bdnf*, *ngf*, *nt3*, *nt4*, *trka*, *full-trkb*, and *trkc* expression was investigated using in situ hybridization (ISH). RNA probes were produced by linearization of TOPO-TA or ZeroBlunt vectors (Invitrogen) containing the amplified polymerase chain reaction (PCR). Antisense and sense riboprobes were generated with DIG RNA Labelling Mix (Roche Diagnostic, Indianapolis, IN, USA) by in vitro transcription, using T7 polymerase (Roche-Diagnostic) and SP6 polymerase (Roche-Diagnostic) on plasmid linearized by EcoRI and Not1. To check the specificity of the staining, the sense and antisense riboprobes were always hybridized on adjacent sections. The following primers were used to generate the riboprobes:
*Ngf*GGAGCACAGGAGATCTACGC and CGTGGAAAAACCCAACTCAT*bdnf-full (fw)*5′-ATAGTAACGAACAGGATGG-3′ and 5′-GCTCAGTCATGGGAGTCC-3′*nt3*ATGGTTACCTTTATTACGATC and CCACCATTTTTCACGTCC*nt4*CAGAGAAGATGCATCCAGGACG and CACTGCCGTTTCCTGACACGCG*trka*AGTTGTTGCTTGCAGGGTGG and TGGGTCAATCATGACCTCAG*trkb-full*CCAGAGATGTGTACAGCACC and CATTGTTTGAGAGCTGATACC*trkc*GGACACTGAGAGCATCTCTATGA and CACGTTGTTCATCCCGACCACATT

Kidneys were quickly removed and fixed overnight at 4 °C in PBS-PAF 4%. Next, they were processed for cryostat. The sections (15 µm) were mounted on slides. They were washed in PBS-NaCl (0.85%) and post-fixed for 20 min in PBS-PFA 4%. The kidney sections were rinsed in PBS and treated for 10 min with proteinase K (2 mg/mL) diluted in PBS at room temperature. Slides were then rinsed as follows: 20 min in 4% PBS-PAF, 10 min in PBS, and 10 min in standard saline citrate (SSC 2x). The sections were incubated overnight at 64.5 °C in a moist chamber with the probes (1.5 µg/mL) diluted in hybridization buffer, as in previous studies [55]. Next, they were dipped in Tris-HCl/NaCl buffer (100 mM, Tris-HCl pH 7.5, 150 mM NaCl) and washed in the same buffer containing 0.1% Triton and 0.5% milk powder. For chromogenic ISH, all sections were incubated overnight at room temperature (RT) with anti-digoxigenin alkaline phosphatase Fab fragments (1:5000, Roche Diagnostic). On the next day, slides were rinsed in Tris-HCl/NaCl buffer and washed three times with Tris-HCl 100 mM (pH 8) containing NaCl (100 mM) and MgCl_2_ (10 mM). Staining was performed with NBT/BCIP buffer (pH 9.5). For fluorescence ISH, all paraffin sections were deparaffinized with ultraclear and rehydrated through a series of graded ethanol (100–30%). Next, the sections were washed and fixed, as mentioned before. In contrast to the chromogenic protocol, the sections were incubated with anti-DIG POD antibody (Roche) at a 1:200 dilution in the above blocking solution overnight at RT. The next day, the sections were washed 4 × 20 min in 1× maleic acid buffer, 4 × 10 min in PBS, and incubated for 1 h in Perkin Elmer amplification diluent buffer. For the reaction, we diluted Cy3 tyramide (TSA plus Cyanine 3, Perkin Elmer, Waltham, MA, USA) reagent 1:100 in amplification buffer and AlexaFluor488 reagent (TSA™ Reagent, Alexa Fluor 488 Tyramide Reagent, Invitrogen) after washing each section three times for 10 min and then observing them with a confocal Nikon Eclipse 90i microscope. The images were cataloged using software (NIS-Elements 4.2).

### 2.5. Immunohistochemistry (IHC)

A total of 5 adult zebrafish kidneys were fixed in Bouin’s solution for 12 h and processed for paraffin embedding. Transverse microtome sections were mounted on poly-lysine slides. Sections were deparaffinized in xylene, rehydrated through graded ethanol, treated with 3% H_2_O_2_ for 30 min, and rinsed in PBS (pH 7.4), followed by antigen retrieval in sodium citrate buffer (pH 6; 80 °C) for 30 min. After rinsing twice in 0.2% Triton PBS (PBT), non-specific binding was blocked by treating sections with 1/5 normal goat serum (Vector, Burlingame, CA, USA; cod S-1000-20) for 30 min at RT. Then, sections were incubated overnight at RT in a humidified chamber with rabbit antibody against NGF, BDNF, NT-3, NT-4, TrkA, TrkB, and TrkC. The antibodies used (see Table 1) have been described in previous studies [61]; we used a dilution of 1/100. The next day, the sections were incubated with the EnVision horseradish anti-peroxidase (HRP) system (Dako, Santa Barbara, CA, USA; cod. K4002).

### 2.6. Protein Analysis by Western Blot

To perform the evaluation of neurotrophins and their receptor proteins, 5 kidneys of adult zebrafish were homogenized using a Tissue Lyzer homogenization system (Qiagen) in 200 μL of ice-cold lysis buffer (50 mM Tris pH 7.5; 150 mM NaCl; 1 mM EDTA; 0.25% deoxicolic acid, 1% Triton X 100) added with 20 mM sodium pyrophosphate, 0.1 mg/mL aprotinin, 2 mM phenylmethylsulphony fluoride (PMSF), 10 mM sodium orthovanadate (Na_2_VO_3_), and 50 mM NaF. The number of total proteins was determined by the use of a protein assay kit (Bio-Rad Laboratories). Equal amounts of lysate samples (60 μg) were boiled and loaded on bis/acrylamide gel 15% (for neurotrophin) or 8% (for Trk receptors) and electrophoresed. All proteins obtained were blotted from the gel onto nitrocellulose membranes. Next, all membranes were blocked with 5% bovine serum albumin (BSA) in Tris-buffered saline (TBS, pH 7.5) containing 0.1% Tween 20 (TBS-T) at room temperature (RT), washed with TBS-T, and incubated with primary antibody (all antibodies are listed in Table 1) diluted 1:500 in TBS-T. After appropriate washing steps in TBS-T, anti-rabbit peroxidase-conjugated secondary antibodies (Amersham, Gel Health Care) were applied for 1 h at RT, at 1:2000 dilution. Finally, the reaction was developed using a chemiluminescent reagent (ECL, Amersham Pharmacia Biotech, Buckinghamshire, UK) and exposed to ChemiDoc gel scanner (Bio-Rad Laboratories). As control, a goat-anti-β-actin antibody (42 kDa) was used. For further details, see Figures S2 and S3.

### 2.7. Microscopy

The stained sections were photographed using a Nikon microscope. The raw digital images were optimized for image resolution, contrast, evenness of illumination, and background by using Adobe Photoshop CS5 (Adobe Systems, San Jose, CA, USA).

## 3. Results

### 3.1. Quantitative Analysis of Neurotrophin and Receptor Expression in Adult Zebrafish Kidney

**Figure 1:**
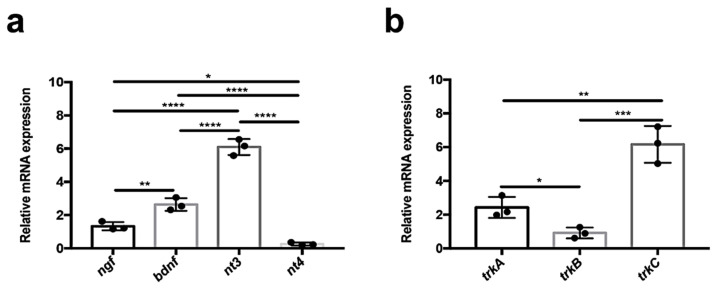
Analysis of neurotrophin and receptor expression by qPCR. (**a**) qPCR analysis for neurotrophic factors: *ngf*; *bdnf*; *nt3*; *nt4*. *ngf* is more expressed than *nt4* (not significatively expressed), but less than *bdnf*. *nt3* is highly expressed compared with previous neurotrophins (* *p* < 0.018; ** *p* < 0.006; **** *p* < 0.0001). (**b**) qPCR analysis for receptors of tyrosine kinase: *trkA*; *trkB*; *trkC*. *trkA* is more expressed than *trkB.* However, *trkC* is highly expressed in the kidney compared with the expression of other receptors (* *p* < 0.020; ** *p* < 0.001; *** *p* < 0.0001).

In order to evaluate neurotrophin and receptor mRNA levels, we performed quantitative PCR experiments in adult zebrafish kidneys. We found that in this organ, *nt3* is highly expressed (compared with the other neurotrophins, *ngf*, *bdnf*). Surprisingly, *nt4* is not significatively expressed (Figure 1a). All receptors of tyrosine kinase are expressed in this organ. In detail, we found abundant expression of receptor *trkc* compared to *trka* and *trkb* (Figure 1b).

### 3.2. Distribution of Neurotrophin and Receptor mRNA in Adult Zebrafish Kidney

**Figure 2:**
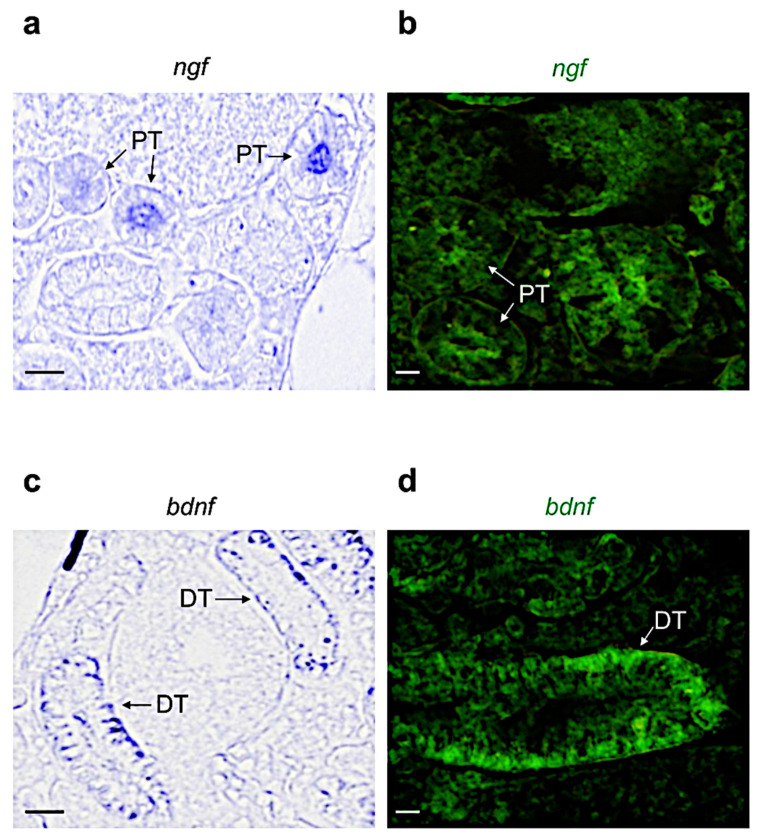
Chromogenic and fluorescence in situ hybridization for *ngf* and *bdnf* on cryostat and paraffin sections of adult zebrafish kidney. (**a**) Chromogenic and (**b**) fluorescence ISH for *ngf-mRNA* in adult zebrafish kidney; it is expressed in proximal tubules (indicated with abbreviation PT). (**c**) Chromogenic and (**d**) fluorescence ISH for *Bdnf*-*mRNA* in adult zebrafish kidney; it is expressed in distal tubules (DT). Scale bars: 100 μm (**a**,**c**); 50 μm (**b**,**d**).

**Figure 3:**
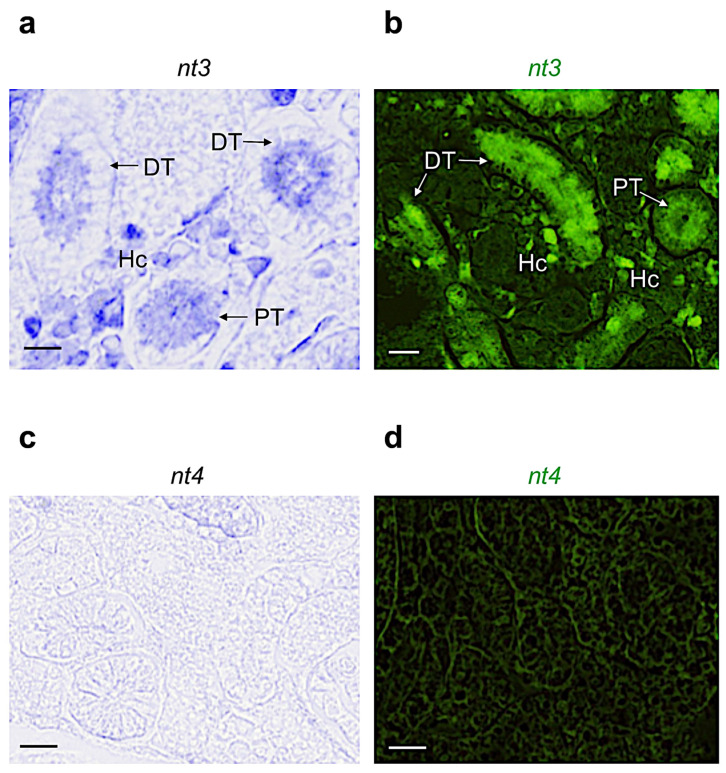
Chromogenic and fluorescence in situ hybridization for neurotrophins *nt3* and *nt4* on cryostat and paraffin sections of adult zebrafish kidney. (**a**) Chromogenic and (**b**) fluorescence ISH for *nt3* in adult zebrafish kidney; it is highly expressed in different regions: proximal and distal tubules, interstitial hematopoietic cells (respective abbreviations: PT, DT, and Hc). (**c**) Chromogenic and (**d**) fluorescence ISH for *nt4*; it is not detected in adult zebrafish kidney. Scale bars: 100 μm (**a**–**d**).

Based on the morphological features described by Menke and colleagues [47], we identified the different regions reported in Figure S1. By using chromogenic and fluorescence in situ hybridization, we confirmed the results obtained by qPCR. In detail, we found that *ngf* is expressed in the proximal tubule (Figure 2a,b). *bdnf* is more expressed in the distal tubule (Figure 2c,d). Differently, *nt3* is highly expressed in the distal and proximal tubules, and in the interstitial hematopoietic cells (by confocal microscopy, we distinguished several shapes that are common to hematopoietic cells: round, tapered, concave, and can present enlarged nucleus) (Figure 3a,b). *nt4*, coherently with qPCR analysis, is not detected in this organ (Figure 3c,d).

**Figure 4:**
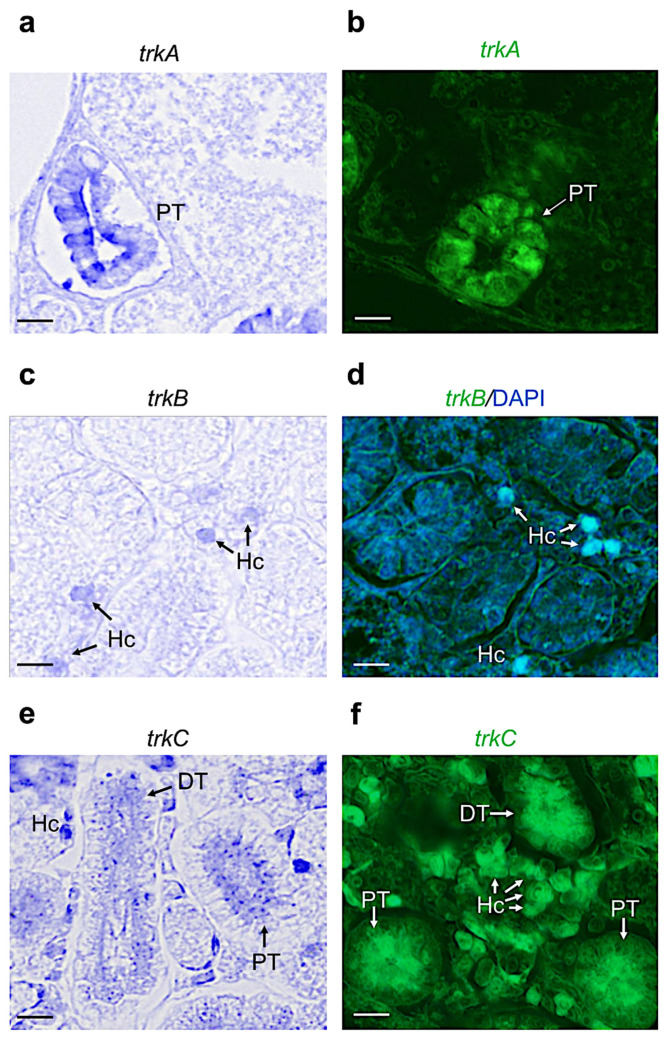
Chromogenic and fluorescence in situ hybridization for tyrosine kinase receptors *trka*, *trkb*, and *trkc* on cryostat and paraffin sections of adult zebrafish kidney. (**a**) Chromogenic and (**b**) fluorescence in situ hybridization of *trkA-mRNA* in adult zebrafish kidney; it is expressed in proximal tubules (PT). (**c**) Chromogenic and (**d**) fluorescence in situ hybridization of *trKB*-*mRNA* and cell nuclei marked with DAPI staining in adult zebrafish kidney; *trkb* is expressed in hematopoietic cells (Hc). (**e**) Chromogenic and (**f**) fluorescence in situ hybridization of *trkC-mRNA*; it is expressed in hematopoietic cells, and proximal and distal tubules (respectively, Hc, PT, and DT). Scale bars: 100 μm (**a**–**f**).

Concerning the receptors, we found that *trkA-*mRNA was expressed in the proximal tubules of the adult zebrafish kidney (Figure 4a,b). *trkb-*mRNA was detected only in a few hematopoietic cells (Figure 4c,d). Finally, *trkc* is highly expressed in hematopoietic cells, and in the distal and proximal tubules (Figure 4e,f).

### 3.3. Expression Levels of Neurotrophin and Receptor Proteins in Adult Zebrafish Kidney

**Figure 5:**
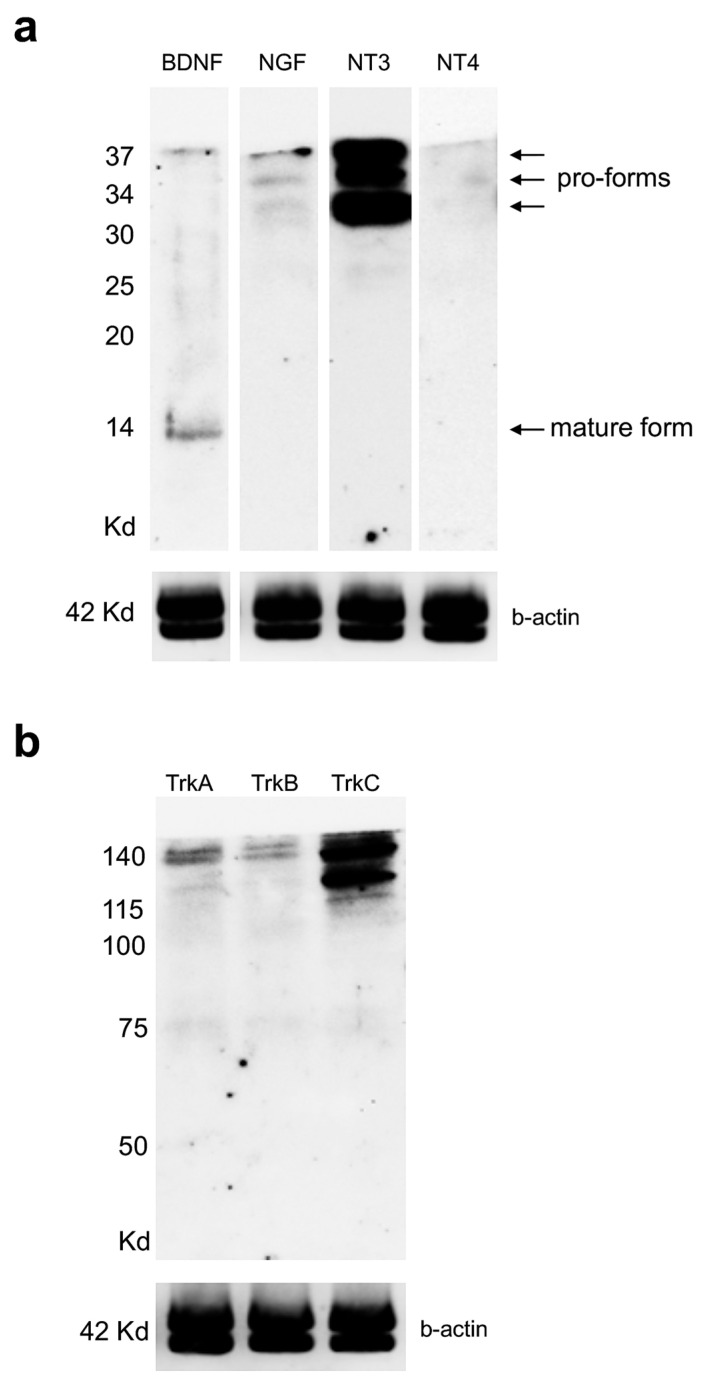
Western blotting analysis for neurotrophins and receptors in adult zebrafish kidney. (**a**) Western blot to detect BDNF, NGF, NT-3, and NT-4 in adult zebrafish kidney. Two bands for BDNF of 14 kD and 37 kD; 3 bands for NGF of 30 kD, 34 kD, and 37 kD; 3 bands for NT-3 of 30 kD, 34 kD, and 37 kD. NT4 is not detected. (**b**) Western blot to detect TrkA, TrkB, and TrkC in adult zebrafish kidney. Three bands for TrKA at 75 kD, 115 kD, and 140 kD. One band of 140 kD for TrkB and 2 bands of 115 kD and 140 kD for TrkC.

Next, to detect the levels of protein for each neurotrophin and receptor in the adult zebrafish kidney, we used Western blot. Interestingly, we found that BDNF antiserum recognized two lines, one at 14 kDa (mature form) and one at 37 kDa (immature form pro-BDNF). NGF antiserum recognized three bands of 30 kDa, 34 kDa, and 37 kDa (these forms are pro-NGF). NT-3 antiserum labeled three bands of 30 kDa, 34 kDa, and 37 kDa (these forms are pro-NT3). NT-4 antiserum was not detected (Figure 5a).

TrkA antiserum showed three bands of 75 kD (low intensity), 115 kD, and 140 kD; TrkB labeled a band of 140 kD and TrkC antiserum recognized two bands of 115 kD and 140 kD (Figure 5b).

### 3.4. Distribution of Neurotrophin and Receptor Proteins in Adult Zebrafish Kidney

**Figure 6:**
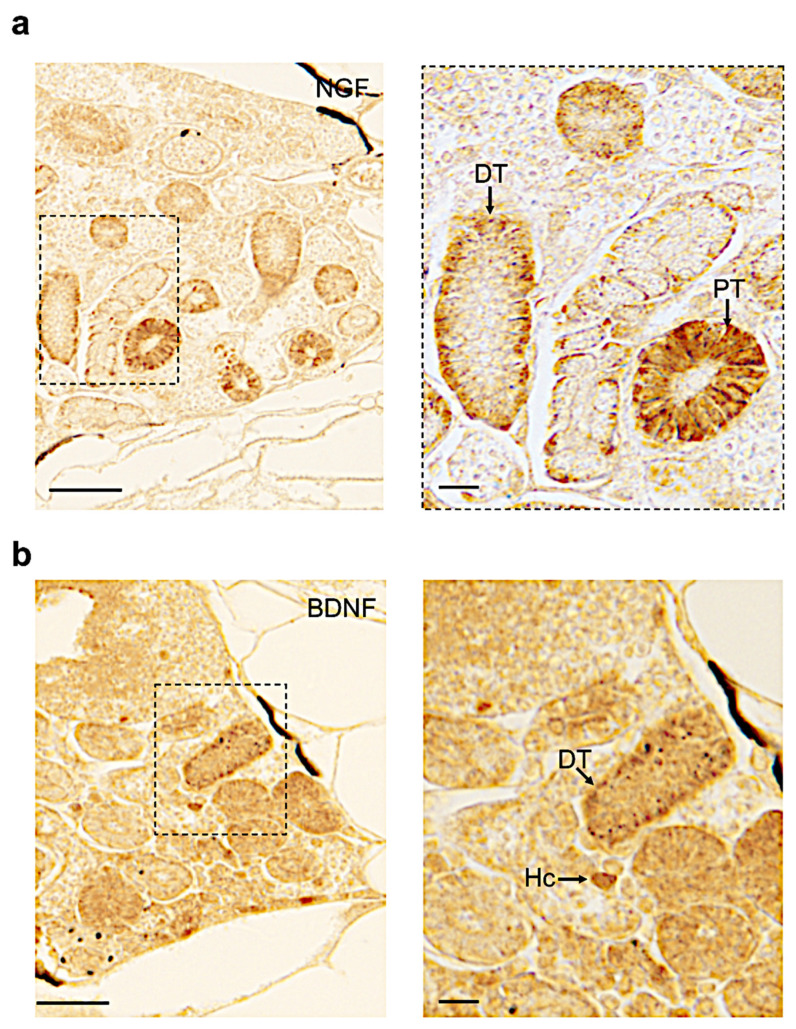
Immunohistochemistry to detect NGF and BDNF on paraffin sections of adult zebrafish kidney. (**a**) NGF protein in adult zebrafish kidney is detected in distal and proximal tubules (respectively, DT and PT). (**b**) BDNF protein is distributed in distal tubules and a few hematopoietic cells (DT and Hc). Scale bars: 200 μm and 50 μm (**a**,**b**).

Finally, by immunohistochemistry, we detected NGF protein in the distal and proximal tubules (Figure 6a). We found BDNF in a few hematopoietic cells and in the distal tubule region (Figure 6b).

**Figure 7:**
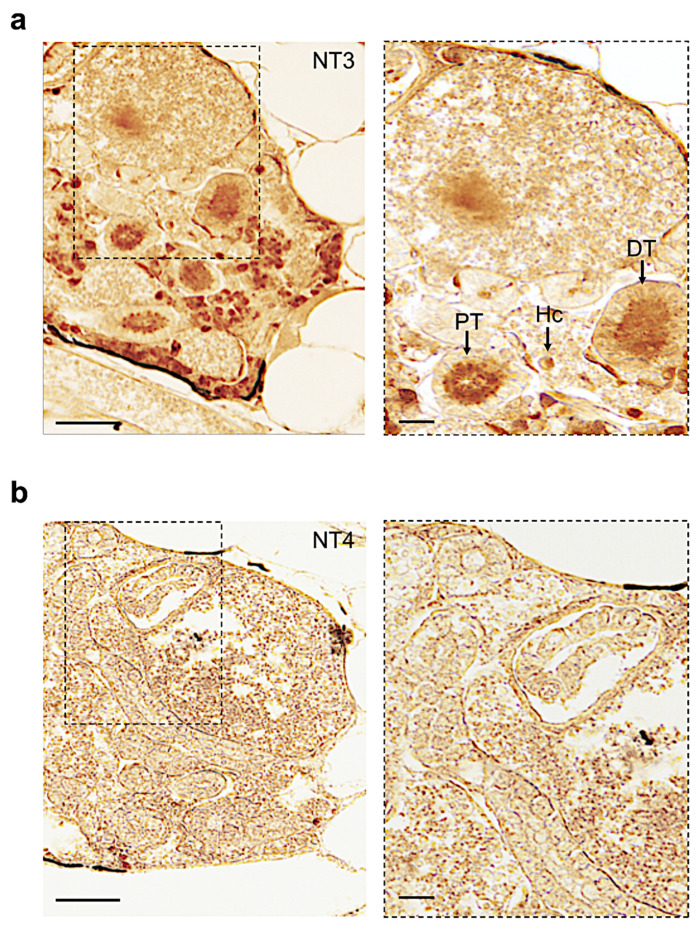
Immunohistochemistry to detect NT-3 and NT-4 on paraffin sections of adult zebrafish kidney. (**a**) NT-3 protein in adult zebrafish kidney is detected in distal and proximal tubules, and hematopoietic cells (DT, PT, and Hc). (**b**) NT-4 protein is not detected. Scale bars: 200 μm and 50 μm (**a**,**b**).

The NT-3 distribution was very abundant in different regions of the kidney (interstitial hematopoietic cells, distal and proximal tubules) (Figure 7a). NT4 was not detected, confirming previous results obtained by Western blot (Figure 7b).

**Figure 8:**
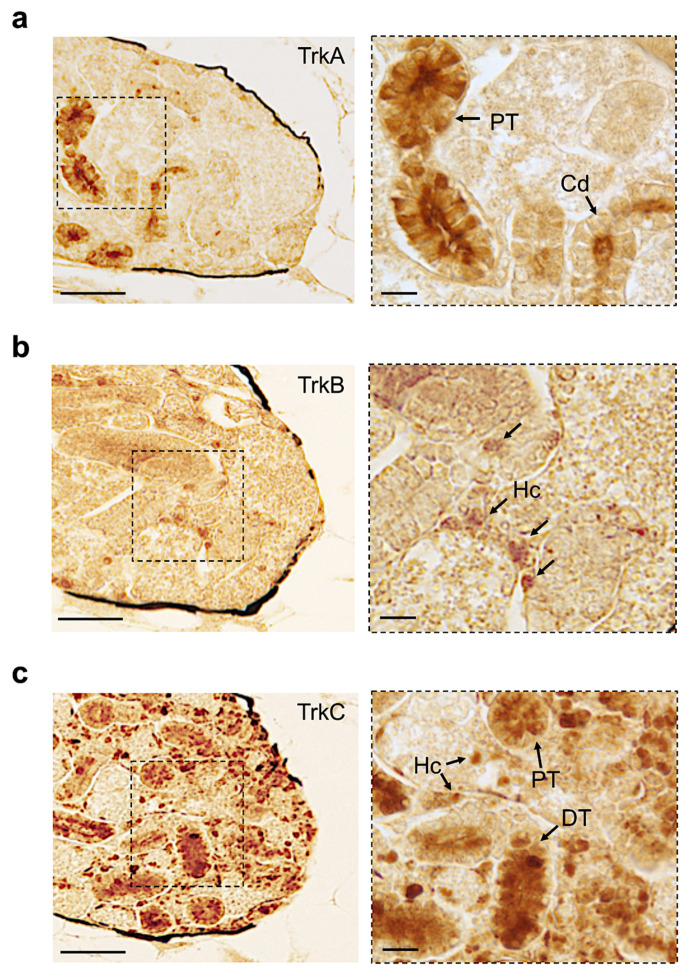
Immunohistochemistry to detect TrkA, TrkB, and TrkC on paraffin sections of adult zebrafish kidney. (**a**) All receptors are expressed in adult zebrafish kidney in different regions. TrkA is expressed in the collecting duct and proximal tubule. (**b**) TrkB is detected in hematopoietic cells. (**c**) TrkC protein is abundantly distributed in different regions: hematopoietic cells; distal and proximal tubules (Hc, DT, and PT). Scale bars: 200 μm and 50 μm (**a**–**c**).

All receptors were expressed in the adult zebrafish kidney in different regions. TrkA was expressed in the collecting duct and proximal tubule (Figure 8a). TrkB was detected in hematopoietic cells (Figure 8b). TrkC was abundantly distributed in different regions. (Figure 8c). We have summarized all our results in the following Table 2.

## 4. Discussion

In the present study, we compared the expression profiles of both mRNA and protein of all neurotrophins/receptors in the kidney of the adult zebrafish.

In this organ, neurotrophin and receptor mRNAs are differently expressed. Our results, obtained by qPCR and ISH, show that *nt3/trkC* is the most expressed in different regions, including the hematopoietic cells, similarly to the mammalian kidney [62,63]. Previous observations in rats and humans highlighted that *nt3* and *trkC* mRNA levels were elevated in the glomerular tissue, and this signaling could be involved in the regulation of renal tubule transport [64,65,66]. In detail, Lepa and colleagues, using a mammal animal model, confirmed that nephron-specific TrkC knockout (TrkC-KO) and TrkC-overexpressing (TrkC-OE) mice presented enlarged glomeruli with mesangial proliferation, podocyte loss during aging, basement membrane thickening, and albuminuria. They also showed that NT3-dependent TrkC activation in cultured podocytes and overexpression of TrkC in the nephron in mice lead to the phosphorylation of the activating tyrosine residues of the Igf1R [66].

Next, we observed that the *ngf* and *trkA* mRNAs were most widely distributed in the proximal tubule, in line with previous reports in the kidneys of humans, mice, and goldfish, where the colocalization of NGF and TrkA was described in the tubular cells [67,68]. Under normal physiological conditions, nerve growth factor and tyrosine kinase receptor-A in humans and mice present low serum levels in the kidney. However, the concentrations can increase during the progression of several inflammatory and autoimmune diseases, such as chronic kidney disease, glomerulonephritis, and after renal transplantation [27,69]. It has been observed that, differently from other tissue, such as the brain, where NGF promoted cell growth, differentiation, and survival, high levels of NGF in the renal tubular cells promote growth arrest and apoptosis [70,71,72,73,74].

More significant is the expression of *bdnf* compared to *ngf*. This result confirms the hypothesis that *bdnf* plays a protective role in the kidney; indeed, low levels of *bdnf* have been detected in an experimental model of chronic nephropathy [28]. Very interestingly, we observed low expression of *trkB* limited to the interstitial hematopoietic cells, in agreement with data reported in the mouse kidney. Indeed, TrkB immunoreactivity, on the other hand, was restricted to the differentiated vascular cells and extraglomerular mesangial cells [75]. *nt4*-mRNA displays a very low level of expression in the kidney of the zebrafish, as also demonstrated in the mouse [63].

With regard to the expression of neurotrophin/receptor proteins, Western blot analysis shows that the employed NGF antiserum recognized three bands, likely corresponding to proNGF proteins, differently from the kidneys of other teleosts, where the mature form of NGF [67] has been detected, and similar to the results obtained in the brain of the teleost in *N. furzeri* and zebrafish [61]. The BDNF antibody detected in two lines (or bands) matches the two isoforms, a first mature BDNF form, and a second immature form—very similar to the data shown in the kidneys of other teleost fish [67]. Pro-BDNF bands have been identified in the zebrafish ovary and testis [20]. NT-3 antiserum identified three bands, presumably pro-NT-3. Previous studies on other teleostean species documented that this antibody detected bands corresponding to the mature form in different tissues [61,67]. Concerning the receptors, the TrkA protein in the kidney is recognized in three bands, and these data have been described in different studies in *Danio rerio* and other teleost species [61,76]. The TrkB antibody detected one band (low level), similar to results reported in other tissue of the zebrafish [22]. The abundant presence of TrkC was recognized in two bands (high level), with a slight difference in the result reported in the brain of the zebrafish [61]. Finally, the immunohistochemical analysis confirms that NT-3 is abundant in the zebrafish kidney, in agreement with reports in the kidneys of goldfish and scorpionfish [67]. NGF and BDNF detection are restricted to the tubule and hematopoietic cells of the zebrafish kidney, differently from that reported in other teleost species [67], and remarkably similar to humans and mice [28,77,78]. NT-4 is not detected, confirming all our previous results obtained by qPCR, ISH, and Western blot. A similar result has been reported in mice [62,63]. In the case of Trk receptors, they were detected in the zebrafish kidney by immunohistochemistry: TrkA was detected in the collecting and proximal tubule, as reported in other teleost species; TrkB was detected only in hematopoietic cells; and TrkC was the most abundant receptor, distributed in different regions.

## 5. Conclusions

This work reports the anatomical distribution of neurotrophins and receptors in adult zebrafish kidneys and also compares the concentrations of mRNA and protein. It is interesting that BDNF and NGF in the zebrafish kidneys are present in tubule and hematopoietic cells, similar to humans and mice. This fact further reinforces the thesis that the zebrafish is an excellent animal model to be used in biomedical and veterinary research. At the same time, our analysis showed that NT3/TrkC is highly expressed in the zebrafish kidney, as also reported in humans and mice. We hope that our work will inspire further experimental studies that could lead to a new potential therapeutic role for neurotrophins and receptors in kidney diseases.

## Figures and Tables

**Table 1:** Antibodies used for immunohistochemistry and Western blotting.

NGF	N-terminus of the mature chain of NGF of human origin	1:100	sc-548 and sc-548P S. Cruz Biotechnology, CA
BDNF	Internal region of BDNF of human origin aa. 100–150	1:100	sc-546 and sc-546P S. Cruz Biotechnology, CA
NT-3	Internal region of NT-3 of human origin	1:100	sc-547 and sc-547P S. Cruz Biotechnology, CA
NT-4	Internal region of NT-4 of human origin	1:100	sc-545 and sc-545P S. Cruz Biotechnology, CA
TrkA	Human COO-domain 763–777 (intracytoplasmic region)	1:100	sc-118 and sc-118P S. Cruz Biotechnology, CA
TrkB	Human COO-domain 794–808 (intracytoplasmic region)	1:100	sc-12 and sc-12P S. Cruz Biotechnology, CA
TrkC	Human COO-domain 798–812 (intracytoplasmic region)	1:100	sc-117 and sc-117P S. Cruz Biotechnology, CA

**Table 2:** Summary of all results obtained by in situ hybridization and immunohistochemistry.

Name	Proximal Tubule	Distal Tubule	Collecting Duct	Hematopoietic Cells
Ngf	+	+	−	−
Bdnf	−	+	−	+
Nt3	+	+	+	+
Nt4	−	−	−	−
Trka	+	−	+	−
Trkb	−	−	−	+
Trkc	+	+	+	+

## Data Availability

Not applicable.

## Supplementary Materials

The following supporting information can be downloaded at: https://www.mdpi.com/article/10.3390/vetsci9060296/s1, Figure S1. Adult zebrafish kidney section HE, available at https://bio-atlas.psu.edu/about.php, accessed on 11 June 2022. Figure S2. Full Western blot gels related to Figure 5a. Figure S3. Full Western blot gels related to Figure 5b.
