# Supplementary material for: Analysis of the Expression of Neurotrophins and Their Receptors in Adult Zebrafish Kidney
Source: Vet Sci. 2022 Jun 15;9(6):296. doi: 10.3390/vetsci9060296 (PMC9227799; doi:10.3390/vetsci9060296)
Supplement: Supplementary file 1 [file vetsci-09-00296-s001.zip › vetsci-1695091-supplementary.pdf]

Adult zebrafish kidney (1 year old)

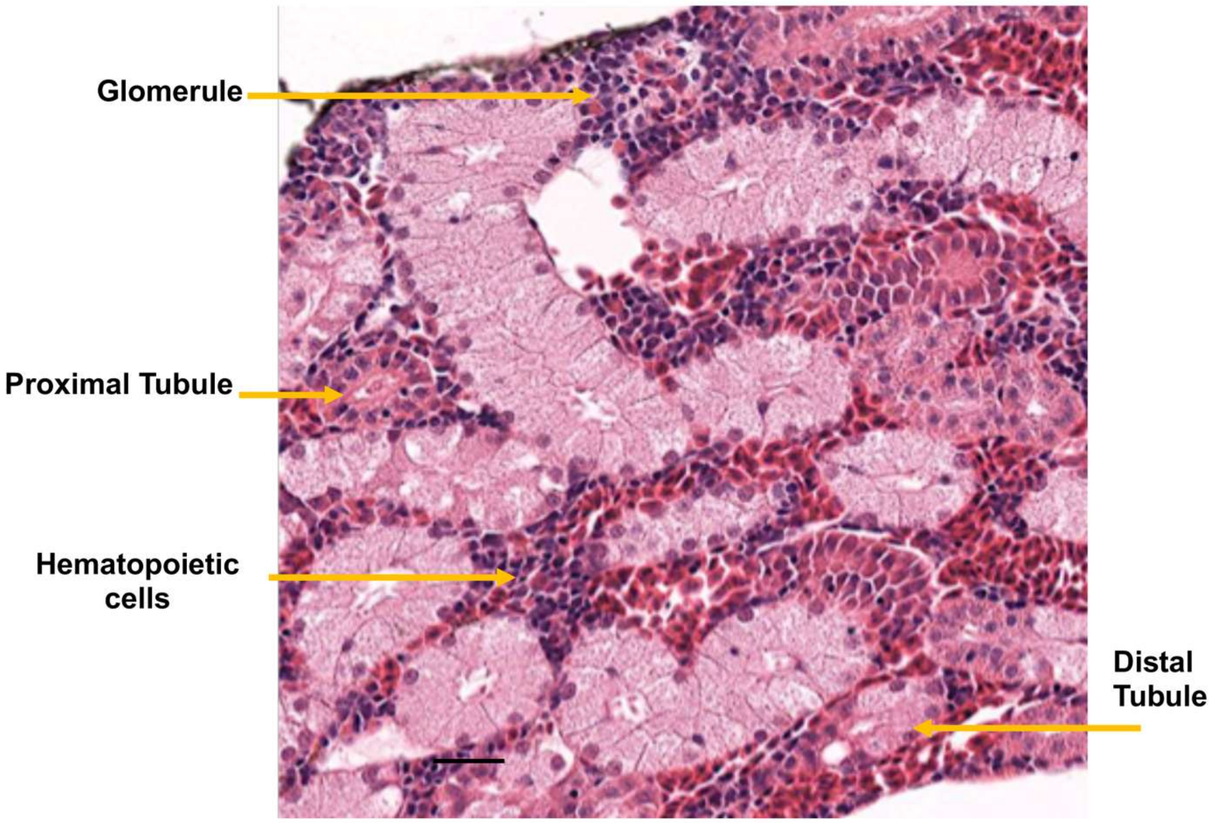

Figure S1. HE staining of zebrafish kidney available in zebrafish atlas. https://bio-atlas.psu.edu/about.php.

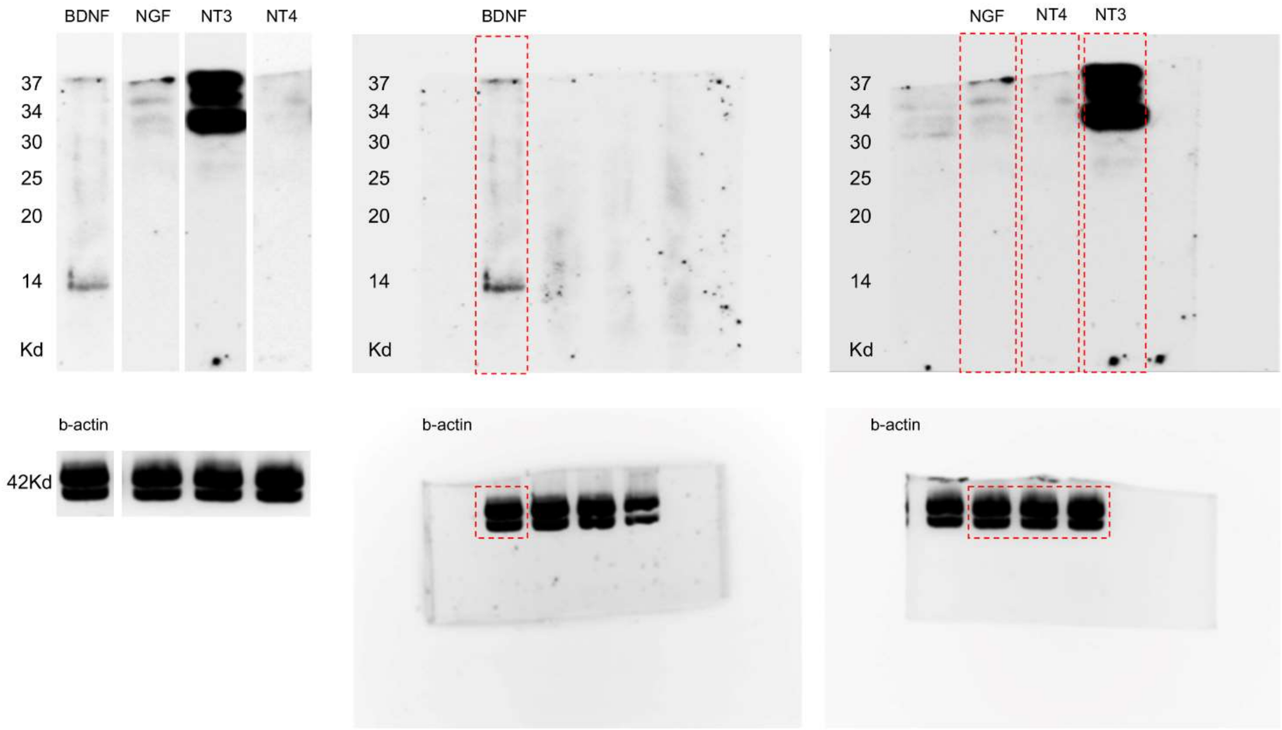

Figure S2. Original WB related to figure 4a.

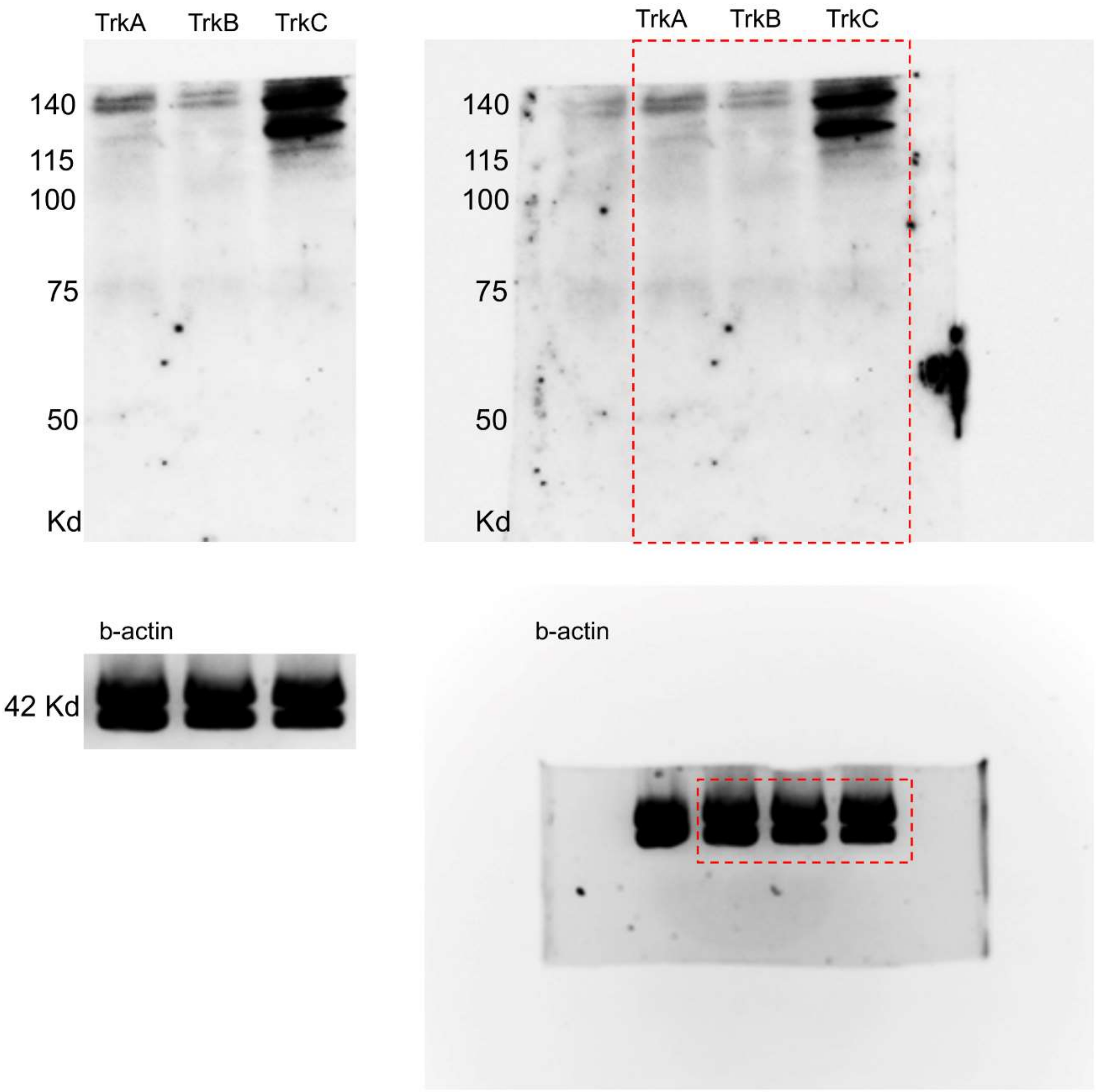

Figure S3. Original Western blotting related to figure 5b.
